# Association of urine phthalate metabolites, bisphenol A levels and serum electrolytes with 24-h blood pressure profile in adolescents

**DOI:** 10.1186/s12882-022-02774-y

**Published:** 2022-04-12

**Authors:** Siddika Songül Yalçin, İzzet Erdal, Berna Oğuz, Ali Duzova

**Affiliations:** 1 Unit of Social Pediatrics, Department of Pediatrics, Hacettepe University Faculty of Medicine, Sihhiye, 06100 Ankara, Turkey; 2 Department of Radiology, Hacettepe University Faculty of Medicine, Ankara, Turkey; 3 Unit of Pediatric Nephrology, Department of Pediatrics, Hacettepe University Faculty of Medicine, Ankara, Turkey

**Keywords:** Adolescence, Phthalates, Mono-benzyl phthalate, Monomethyl phthalate, Bisphenol A, Calcium, Carotid intima-media thickness, Ambulatory blood pressure monitoring, Nocturnal hypertension

## Abstract

**Background:**

Among the possible causes of hypertension in adolescence, electrolyte imbalances and environmental pollutants are drawing increasing attention. We aimed to examine the relationship between bisphenol A (BPA), phthalate metabolites, and serum electrolytes and blood pressure.

**Methods:**

Eighty-six participants aged 12–15 years were included in the study. Body mass index (BMI), office blood pressure and 24-h ambulatory blood pressure measurements (ABPM), and carotid intima-media thickness were determined. Blood samples were taken for hemogram, renal function tests, and serum electrolytes. Free- and total-BPA and phthalate metabolites were analyzed from urine samples.

**Results:**

Of the participants, 34 were evaluated as normal blood pressure profile, 33 as white-coat hypertension (WCHT), and 19 as ABPM-hypertension. Adolescents in ABPM- hypertension groups had higher BMI-standard deviation score (SDS), leucocyte, platelet count; but lower serum chloride, compared to the normal blood pressure profile group. The percentage of adolescents with detectable urinary mono-benzyl phthalate (MBzP) was higher in ABPM-hypertension (42.1%) and WCHT groups (33.3%), compared to the normal blood pressure profile group (5.9%, *p* = 0.004). Associations between MBzP and ABPM- hypertension and WCHT were remained after confounding factor adjustment. Adolescents with detectable MBzP levels had also higher “albumin-corrected calcium” and lower serum phosphate and “albumin-corrected calcium x phosphate product” compared to others. Adolescents with detectable urinary MBzP levels had higher blood pressure profiles in some 24-h (mean arterial pressure-SDS, systolic blood pressure-SDS), daytime (systolic blood pressure-SDS), and night-time (mean arterial pressure-SDS, systolic blood pressure-SDS, and diastolic blood pressure-SDS) measurements, compared to others. WCHT was found to be associated negatively with monomethyl phthalate and the sum of dibutyl phthalate metabolites and ABPM-HT with MCPP. There was no significant association between blood pressure profiles and free- and total-BPA status.

**Conclusion:**

MBzP was associated with adverse blood pressure profiles in adolescence. Additive follow-up studies are necessary for cause-effect relations.

**Supplementary Information:**

The online version contains supplementary material available at 10.1186/s12882-022-02774-y.

## Introduction

The worldwide increase in the incidence of hypertension (HT) and obesity in the pediatric population have critical long-term cardiovascular effects [1]. Studies suggest that primary HT may be associated with inflammation, hemodynamic and metabolic changes in the body related to inadequate or excessive micro- and macronutrient intake and exposure to pollutants in addition to obesity, race/ethnicity, physical inactivity, and poor sleep quality [2–7].

Calcium, phosphate, magnesium, sodium, potassium, and chloride have crucial roles in patients with various diseases, and even in the general population [7–10]. High serum calcium level was reported to be associated with metabolic syndrome, diabetes, and HT among adults from Taiwan [8]. Low serum phosphate has been shown to be related with metabolic syndrome, HT, increased sympathoadrenal activity [11, 12]. However, controversial results were reported in serum chloride levels [10, 13].

Phthalates, one of the man-made pollutants, are environmental chemicals that are widely used in consumer goods and personal care products today. Since these substances are not covalently bonded to the polymer, they can affect the environment and people by mixing with the atmosphere, food product, or directly into body fluids in various ways [14]. Phthalates may have toxic effects on the immunological, endocrine, cardiovascular systems, renal functions, and development [2, 15–19]. Although there are studies on the relationship between phthalate metabolites and blood pressure, very few of them have been done in children [2, 20, 21]. However, the studies in children did not evaluate the relationship between phthalates and changes in 24-h blood pressure up to now.

Since bisphenol A (BPA) is widely used in the manufacture of polycarbonate plastics, epoxy resins and thermal paper, it is found in many products such as plastic bags, water bottles, toothpastes, electronic equipment, paper and toys [22]. In addition to BPA being one of the endocrine disruptors, studies are showing its hepatotoxic, immunotoxic, obesity-forming, and carcinogenic effects [19, 23]. The results of the few studies conducted in the pediatric age group on the relationship of BPA with cardiovascular diseases are inconsistent [24–27].

Previous studies evaluated the effects of pollutants such as BPA and phthalates on the cardiovascular system or kidney diseases, regardless of the micronutrient balances of the cases [2, 16–18, 20]. However, there might be an interaction between the level and metabolism of pollutant exposure and micronutrient status [28–31]. There is no study examining the influence of exposure to BPA and phthalates and essential electrolyte balance on blood pressure, simultaneously. In addition, the interaction between free-BPA and blood pressure is unknown.

Most studies about HT were performed by office blood pressure measurement. However, ambulatory blood pressure monitoring (ABPM) has a higher reproducibility and a better correlation with end-organ damage, compared to office blood pressure [32]. Therefore, ABPM provides a better assessment of blood pressure with a studied subject.

We aimed to investigate how serum electrolytes and urinary levels of 14 phthalate metabolites and BPA, including free and total are associated with (a) blood pressure profiles [white-coat HT (WCHT), HT, sustained, daytime, night-time], and (b) carotid intima-media thickness (cIMT) in a group of asymptomatic adolescents who are not on antihypertensive medication. Understanding the relation between serum electrolytes, pollutants and blood pressure may be important to define risk groups for HT and to take preventive measures. The detection of possible associations for HT and management of these factors during childhood period could be important to prevent cardiovascular disease in adolescents and adults.

## Methods

The target population of the study was adolescents aged 12–15 years who were evaluated in the Child Health Checkup Study (CHCS) at the Hacı Sabancı Secondary School and Ticaret Odası Secondary School between December 2017 and March 2018. Adolescents who were newly diagnosed with HT in CHCS and following healthy growing age-appropriate (±1 years) adolescents were enrolled for the study. Exclusion criteria included any cardiac, renal or endocrine disorders, and taking any medicine that can alter blood pressure.

The study protocol was approved by the Provincial Directorate of National Education and Hacettepe University Ethics Committee (GO:16/582). Written informed consent was obtained from the participating children and their families before enrollment in the study.

All participants’ physical examinations including office blood pressure and anthropometric measurements (height and weight) were taken as part of the CHCS. With the WHO-Anthro Plus database, standard deviation scores (SDS) of height and body mass index (BMI) were estimated [33]. Obesity was defined as BMI-SDS ≥ 2. Office- blood pressure measurements were taken with an electronic blood pressure monitor (Omron MIT Elite plus, Dalian, China). During the measurement, the adolescents sit on their backs and put their feet on the ground at a 90-degree angle for 5 min, and the arm was supported at heart level. Two measurements were taken one week apart, and adolescents with systolic blood pressure and/or diastolic blood pressure persistently equal to or greater than 95th percentile (based on age, sex, and height percentiles published by the AAP) [34] were defined as office-HT. After each case with office-HT, one adolescent having normal blood pressure on two measurements was considered for control cases.

Cases with high blood pressure and control cases were called to the hospital for further evaluation. From adolescents admitted to hospital, age, sex, history of diseases and medication usage, and parental age and HT were taken with a questionnaire. Then, venous blood and urine samples were taken on admission to the hospital, and 24-h ABPM and ultrasonography for cIMT were performed the next day.

A venous blood sample was taken into EDTA containing tube for complete blood count [hemoglobin, white blood cell (WBC), platelets] and plain tube for protein, albumin, urea nitrogen, creatinine, sodium, potassium, chloride, calcium, magnesium. Then, urine samples were taken into glass containers and divided into two 10-mL aliquots into amber glass vials and tops packed with aluminum foil; and one stored at − 20 °C until analyses for phthalates metabolites and BPA levels. The second aliquot was used for urinary creatinine, albumin, protein, and beta-2 microglobulin. Laboratory analyses were performed at a reference laboratory with appropriate kits.

Albumin-corrected calcium (ACCa) was defined as the formula given below.

$$\mathrm{ACCa}=\mathrm{total}\ \mathrm{calcium}\ \left(\frac{\mathrm{mg}}{\mathrm{dl}}\right)+\left[4.0-\mathrm{serum}\ \mathrm{albumin}\ \left(\frac{\mathrm{g}}{\mathrm{dl}}\right)\right]\times 0.8$$

Urinary albumin, protein, and beta-2 microglobulin levels were adjusted for urinary creatinine. Estimated glomerular filtration rate (eGFR) was calculated with Schwartz formula [35].

### Ambulatory blood pressure monitoring (ABPM)

ABPM measurements were made from the patient’s non-dominant arm with the cuff of the appropriate size and the Spacelabs Monitor 90,207 device (Spacelabs Healthcare, Snoqualmie, Washington, USA). Measurements were made every 15–20 min during the waking period and 30 min during the sleep period; ABPM data were evaluated with a measurement time of at least 18 h without interruption; at least 20 measurements during wakefulness and at least 7 measurements during sleep; at least one measurement was made per hour, including the sleep period [36, 37]. SDS for systolic blood pressure, diastolic blood pressure and mean arterial pressure (MAP) were estimated with LMS values according to age, height, and gender [38].

**Fig. 1:**
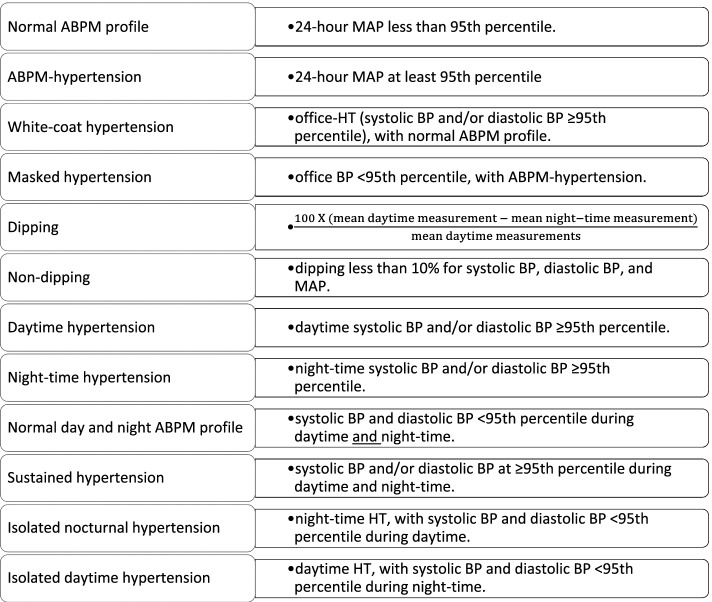
Definitions for ABPM profiles

*Definitions of ABPM profiles including n*ormal ABPM profile, ABPM-HT, WCHT, masked HT and dipping were given in Fig. 1. ABPM profiles were further evaluated for the presence of daytime (from 08.00 am to 08.00 pm), night-time (from midnight to 06:00 am), and sustained HT (Fig. 1) [38, 39].

### Carotid intima-media thickness (cIMT)

Ultrasound examination for cIMT measurement was performed with an Affiniti 70G ultrasound system (Philips Medical Systems, Holland) using 5-18 MHz linear probe according to Mannheim consensus [40]. Five measurements were taken and the average was converted to SDS values using standard LMS values for gender and height [41].

### Bisphenol A (BPA)

Free BPA (fBPA) and total BPA (tBPA) levels were measured using the MyBioSource Human fBPA ELISA kit (Cat no: MBS047388, Southern California, SanDiego) and the Human tBPA ELISA Kit (Cat no: MBS109499) by the user manual.

### Analysis of phthalate metabolites

Of phthalates, 14 metabolites [mono-n-butyl phthalate (MnBP), mono-benzyl phthalate (MBzP), monocarboxy isononyl phthalate (MCiNP), mono carboxy isooctyl phthalate (MCiOP), mono 3-carboxypropyl phthalate (MCPP), mono 2-carboxymethylhexyl phthalate (MCMHP), mono 2-ethyl-5-carboxypentyl phthalate (MECPP), mono 2-ethylhexyl phthalic acid (MEHP), mono 2-ethyl 5-hydroxyhexyl phthalate (MEHHP), mono 2-ethyl-5-oxy-hexyl phthalate (MEOHP), monoethyl phthalate (MEP), monoisobutyl phthalate (MiBP), monoisononyl phthalate (MiNP), monomethyl phthalate (MMP)] were explored in urine sample using the ‘Waters ACQUITY UPLC-MS/MS System (Waters Acquity, the United States/Milford)’ device using the Acquity UPLC BEH Phenyl Column [42, 43] in Certificated Reference Laboratory (Düzen Norwest Environmental Laboratory, Ankara, Turkey). UPLC-MS/MS system was operated under suitable conditions [the capillary voltage: 3 kV, desolvation temperature: 250 °C, ion source temperature: 150 °C, cone gas: 50 L/h, desolvation gas flow: 600 L/h] according to the manufacturer’s instructions. Details on the analytic methods for the phthalates have been previously [44]. Briefly, urine samples were processed using enzymatic deconjugation and extracted, and analysis was performed. The limit of detection (LOD) was below 0.3 μg/L for all studied metabolites (Suppl Table 1). Quality control (QC) samples were included in every 10 analyzed samples and each analytical run. Recovery rates for phthalate biomarkers ranged from 90.0 to 112.8. Validation of results showed the high accuracy of the method and given in Supplementary Table 1.

The determination of urinary creatinine (Cre) levels was based on Jaffe’s colorimetric method with an automatic biochemical analyzer to correct urine dilution (Beckman Coulter ORS61780 21). The concentrations of urinary phthalate metabolites and BPA were determined as both unadjusted (crude; μg/L) and creatinine corrected values (μg/g-cre).

ΣDEHP [the sum of di (2-ethylhexyl) phthalate (DEHP) metabolites] was estimated by adding the molar concentrations of five metabolites: MEHP (MW = 278), MEHHP (MW = 294), MEOHP (MW = 292), MECPP (MW = 308) and MCMHP (MW = 308) and multiplied by the MW of MECPP [44, 45]. The sum of the dibutyl phthalate (DBP) metabolites (ΣDBP) was calculated by adding the molar concentrations of two metabolites: MiBP and MnBP and multiplied by the MW of MnBP [46].

Primary/secondary DEHP metabolites calculated by dividing the molar concentration of MEHP by the sum of MEHHP, MEOHP, MECPP, and MCMHP.

### Statistical analysis

Detectable rates were fBPA 12.8, MBzP 24.4%, MEP 95.3%, MEHP 97.7%, MCINP 77.9%, MCPP 80.2%, MMP 89.5%, MiNP 11.6% (Supplementary Table 2). Parameters having low detection ratios (< 80%) were only compared with the Chi-square test. Frequencies of cases having the median levels or higher for metabolites in groups were also compared.

The distribution of data in groups was evaluated with Shapiro-Wilk, kurtosis, skewness, and histograms. BPA and phthalate metabolites are skewed and compared with the Mann Whitney U-test for two groups and Kruskal-Wallis for three groups. Subgroup analysis in three groups was performed with pairwise comparisons adjusted by Bonferroni correction. Parameters with homogeneous distribution were compared with the Student t-test or ANOVA as appropriate. Subgroup analysis was performed with Duncan. All statistical analyses were performed both with crude and creatinine-adjusted phthalate concentrations. Data are presented as %, or median (Q1-Q3) or mean and standard deviation as appropriate. Urinary phthalate metabolites having *p* value less than 0.20 in univariate analysis for ABPM profiles were taken for further analysis.

Multinominal logistic regression analyzed the differences in rates of selected urinary phthalate metabolites having ≥ median value or detectable levels among groups of blood pressure profiles (WCHT vs. normal; ABPM hypertension vs. normal) with four models. Model 0 was crude model. Model 1 included adjustment for age, sex, BMI-SDS. Model 2 included WBC, platelet, Cl, alb-adj-CaXP, eGFR, U-cre in addition to Model 1. In addition to parameters of Model 2, Model 3 covered five phthalate metabolites having *p* value < 0.2 for either WCHT or ABPM hypertension in Model 2.

Logistic regression were used for the differences in rates of urinary phthalate metabolites having ≥ median value or detectable levels among groups of “day or/and night HT” vs normal profile with four models. Model 0 was crude model. Model 1 included adjustment for age, sex, BMI-SDS and parental HT. Model 2 included platelet, eGFR, U-cre in addition to Model 1. Model 3 covered phthalate metabolites having p value < 0.2 with confounding factors in Model 2. Besides, cases were divided into 4 groups according to MEP (≥ or < median value) and MBzP (detectable or not) and association with day-time and/or night-time HT were evaluated with logistic regression.

The association of the rates of the high ΣDEHP metabolites with cIMT SDS higher than 95th percentile were analyzed with logistic regression in crude model, after adjustment for age, sex and BMI-SDS, and after controlling for age, sex, BMI-SDS, eGFR, U-cre.

The Odds ratio (OR) and 95% confidence interval (CI) were calculated for logistic regressions.

SPSS version 22 (SPSS, Inc., Chicago, USA) was used for statistical analyses. *P*-values < 0.05 were accepted as statistically significant.

## Results

After repeated office blood pressure measurements 57 adolescents with office HT and 46 adolescents with normal office blood pressure were admitted to the hospital for further evaluation. Three had Hashimoto thyroiditis, two did not give urine and four had no ABPM and eight had attention deficit hyperactivity disorders and they were excluded from the study. At the end, 48 adolescents with office HT and 38 adolescents with normal office blood pressure completed the study. Following 24-h ABPM, adolescents were classified as normal office blood pressure and ABPM (normal blood pressure profile, *n* = 34), WCHT (*n* = 33) and ABPM-HT (*n* = 19). Since the number of adolescents with masked-HT was low, they were not analyzed separately and were included in the ABPM-HT group; i.e. ABPM-HT included adolescents with office and ABPM-HT (*n* = 15) and masked-HT (*n* = 4).

Clinical characteristics, laboratory findings, and distribution of urinary BPA and phthalate metabolites according to blood pressure groups based on office blood pressure and ABPM are shown in Table 1. The groups were comparable for age, sex, and height-SDS. Adolescents in the ABPM-HT group had higher BMI-SDS, WBC, platelet count; but lower serum chloride, compared to the normal blood pressure profile group. In addition, adolescents with WCHT had lower WBC, platelet count, calcium, ACCa levels, compared to ABPM-HT group; with lower chloride and ACCa-phosphate product, compared to the normal blood pressure profile group. Carotid IMT-SDS was higher in WCHT, compared to the normal blood pressure profile group; but the difference between the ABPM-HT and the normal blood pressure profile group did not reach to statistical significance.

**Table 1:** Clinical characteristics and laboratory findings of adolescents according to blood pressure groups based on office and ambulatory blood pressure monitoring

Parameters	Blood pressure groups			p	ABPM groups			p
	Normal BP profile (*N* = 34)	WCHT (*N* = 33)	ABPM-HT (*N* = 19)		Normal day-time and night-time BP (*N* = 61)	Daytime *or* night-time HT (*N* = 13)	Sustained (daytime *and* night-time) HT (*N* = 12)	
Clinical characteristics								
Female/male, % / %	44.1/55.9	54.5/45.5	52.6/47.4	0.672	42.5/47.5	61.5/38.5	25.0/75.0	0.147
Age (years)	13.3 ± 0.7	13.4 ± 0.7	13.2 ± 0.8	0.772	13.3 ± 0.7	13.4 ± 0.7	13.1 ± 0.9	0.495
Height-SDS	0.39 ± 1.19	0.73 ± 0.81	0.64 ± 0.74	0.344	0.52 ± 1.06	0.75 ± 0.71	0.67 ± 0.76	0.692
BMI-SDS	0.51 ± 1.29^a^	1.02 ± 1.12^ab^	1.45 ± 1.10^b^	0.019	0.65 ± 1.22^a^	1.37 ± 0.82^ab^	1.76 ± 1.17^b^	0.005
Obesity, %	14.7	24.2	31.6	0.340	16.4	30.8	41.7	0.111
Parental characteristics								
Mother’s age (years)	41.2 ± 6.7	40.4 ± 6.3	40.4 ± 4.3	0.865	40.7 ± 6.1	42.3 ± 7.3	39.3 ± 4.2	0.478
Father’s age (years)	45.5 ± 7.2	46.6 ± 7.6	42.1 ± 3.9	0.088	45.1 ± 7.2	48.3 ± 6.2	42.0 ± 4.9	0.084
Parental hypertension, %	11.8	24.2	31.6	0.197	14.8^a^	23.1^ab^	50.0^b^	0.023
Laboratory findings								
Hemoglobin (g/dl)	14.2 ± 1.1	14.2 ± 0.9	13.9 ± 1.0	0.572	14.1 ± 1.0	14.2 ± 1.3	14.0 ± 0.9	0.887
White blood cell (×10^3^/mm^3^)	7.1 ± 1.8^a^	7.4 ± 1.8^a^	9.2 ± 2.9^b^	0.002	7.3 ± 2.0	8.7 ± 3.0	8.3 ± 1.9	0.073
Platelets (×10^3^/mm^3^)	272 ± 44^a^	267 ± 65^a^	318 ± 67^b^	0.006	271 ± 58^a^	287 ± 66^ab^	320 ± 60^b^	0.036
Serum protein (g/dl)	7.5 ± 0.4	7.7 ± 0.4	7.8 ± 0.5	0.051	7.6 ± 0.4	7.6 ± 0.3	7.8 ± 0.5	0.336
Serum albumin (g/dl)	4.6 ± 0.3	4.6 ± 0.3	4.7 ± 0.3	0.792	4.6 ± 0.3	4.7 ± 0.3	4.7 ± 0.3	0.370
Serum globulin (g/dl)	2.9 ± 0.3	3.0 ± 0.2	3.1 ± 0.5	0.104	3.0 ± 0.3	2.8 ± 0.2	3.0 ± 0.4	0.246
Blood urea nitrogen (mg/dl)	11.8 ± 2.8	10.5 ± 2.6	11.2 ± 3.1	0.195	10.9 ± 2.8	12.6 ± 3.3	11.0 ± 2.4	0.163
Creatinine (mg/dl)	0.56 ± 0.11	0.59 ± 0.11	0.53 ± 0.08	0.127	0.57 ± 0.11	0.58 ± 0.12	0.55 ± 0.07	0.787
eGFR [Schwartz] (ml/min/1.73m^2^)	121.4 ± 19.8	117.3 ± 19.0	128.0 ± 19.5	0.165	121.1 ± 20.1	121.6 ± 24.0	122.1 ± 12.1	0.986
Uric acid (mg/dl)	5.0 ± 1.2	5.2 ± 1.1	5.3 ± 1.1	0.564	5.0 ± 1.2	5.0 ± 1.0	5.6 ± 0.9	0.219
Sodium (mEq/L)	139.4 ± 1.0	138.8 ± 1.8	138.4 ± 1.5	0.066	139.0 ± 1.5	139.2 ± 1.2	138.2 ± 1.6	0.162
Potassium (mEq/L)	4.3 ± 0.2	4.1 ± 0.3	4.2 ± 0.3	0.078	4.2 ± 0.3	4.2 ± 0.2	4.3 ± 0.3	0.384
Chloride (mEq/L)	105.0 ± 1.8^a^	103.8 ± 2.0^b^	103.5 ± 2.2^b^	0.016	104.5 ± 1.9	103.7 ± 1.7	103.3 ± 2.6	0.106
Calcium (mg/dl)	9.9 ± 0.4^ab^	9.7 ± 0.3^a^	10.0 ± 0.4^b^	0.026	9.8 ± 0.4	10.0 ± 0.3	10.0 ± 0.4	0.106
ACCa (mg/dl)	9.4 ± 0.3^ab^	9.2 ± 0.3^a^	9.5 ± 0.3^b^	0.015	9.3 ± 0.3	9.4 ± 0.3	9.4 ± 0.4	0.319
Phosphate (mg/dl)	4.6 ± 0.6	4.3 ± 0.6	4.2 ± 0.6	0.051	4.4 ± 0.6	4.3 ± 0.6	4.3 ± 0.6	0.759
ACCa-phosphate product (mg^2^/dl^2^)	43.0 ± 6.0^a^	39.5 ± 5.8^b^	40.0 ± 5.8^ab^	0.045	41.2 ± 6.1	40.8 ± 5.3	40.4 ± 6.8	0.925
ACCa-phosphate ratio (mg/mg)	2.01 (1.86–2.30)	2.14 (1.94–2.39)	2.25 (2.01–2.49)	0.069	2.10 (1.90–2.36)	2.12 (1.96–2.38)	2.19 (1.93–2.53)	0.661
Magnesium (mg/dl)	2.07 ± 0.12 (*n* = 31)	2.04 ± 0.16 (*n* = 24)	1.99 ± 0.16 (*n* = 12)	0.297	2.05 ± 0.14	2.01 ± 0.17	2.03 ± 0.13	0.752
UPCR (mg/mg)	0.09 (0.07–0.12)	0.08 (0.07–0.11)	0.08 (0.07–0.10)	0.450	0.08 (0.07–0.11)	0.08 (0.06–0.11)	0.09 (0.07–0.12)	0.933
UACR (mg/g)	7.0 (3.2–18.4)	6.0 (4.6–15.5)	7.6 (5.2–14. 6)	0.951	7.1 (4.0–15.8)	6.1 (3.5–14.3)	6.9 (4.8–26.9)	0.751
UBCR (μg/g)	57.7 (25.8–73.8)	58.5 (19.6–89.1)	43.4 (21.7–60.1)	0.430	57.9 (21.2–74.6)	65.8 (32.9–93.7)	42.2 (18.9–59.8)	0.376
Carotid IMT-SDS	0.84 ± 1.26^a^	1.73 ± 1.10^b^	1.43 ± 0.95^ab^	0.008	1.26 ± 1.27	1.67 ± 0.99	1.16 ± 0.92	0.499
Carotid IMT-SDS ≥95 percentile, %	24.2	53.1	38.9	0.057	38.3	38.5	40.0	0.995

Data are presented as % or mean ± standard deviation or median (Q1-Q3) as appropriate

^ab^Values having different letter were statistically significant

*ABPM* ambulatory blood pressure monitoring, *ACCa* albumin-corrected calcium, *BMI* body mass index, *BP* blood pressure, *eGFR* estimated glomerular filtration rate, *HT* hypertension, *IMT* intima media thickness, *SDS* standard deviation score, *UACR* urinary albumin to creatinine ratio, *UBCR* urinary beta-2 microglobulin to creatinine ratio, *UPCR* urinary protein to creatinine ratio, *WCHT* white coat hypertension

On the other hand, WBC and platelet count in both normal blood pressure profile and WCHT groups were remained to be significantly lower than that the ABPM-HT group when controlled for BMI-SDS (for WBC count, mean ± SEM = 7.3 ± 0.4, 7.4 ± 0.4, 9.0 ± 0.5 × 10^3^/mm^3^, respectively, *p* = 0.011 and for platelet count, 281 ± 9, 264 ± 9, 308 ± 13 × 10^3^/mm^3^, respectively, *p* = 0.023).

The differences for urinary levels of tBPA and other phthalate metabolites according to blood pressure groups did not reach to statistical significance (Table 2). The percentage of fBPA equal to or greater than detectable level was slightly higher in ABPM-HT (15.8%) and WCHT groups (21.2%), compared to the normal blood pressure profile group (3.0%, *p* = 0.081).

**Table 2:** Levels of urinary bisphenol A (BPA) and phthalate metabolites (uncorrected and corrected for urinary creatinine) of adolescents according to blood pressure groups

Parameters		Blood pressure groups			p	ABPM groups			
		Normal BP profile	WCHT	ABPM-HT		Normal day-time and night-time BP	Daytime *or* night-time HT	Sustained (daytime *and* night-time) HT	p
n		34	33	19		61	13	12	
Total BPA	μg/g-cre	79 (46–161)	115 (68–153)	88 (49–122)	0.397	85 (53–156)	123 (82–192)	92 (44–127)	0.410
	μg/L	125 (83–185)	142 (119–189)	169 (87–182)	0.600	128 (85–176)	160 (125–203)	165 (88–203)	0.273
Free BPA	≥DL, %	3.0	21.2	15.8	0.081	13.1	7.7	16.7	0.790
**High molecular weight phthalate metabolites**									
MCiNP	≥DL, %	91.2^a^	75.8^ab^	57.9^b^	0.018	85.2^a^	46.2^b^	75.0^ab^	0.008
	μg/g-cre	0.66 (0.50–1.00)	0.47 (0.05–0.85)	0.43 (<DL-1.15)	0.199	0.62 (0.42–0.91)	<DL (<DL-0.93)	0.65 (0.06–1.70)	0.176
	μg/L	1.08 (0.70–1.49)	0.79 (0.05–1.10)	0.86 (<DL-1.83)	0.126	0.87 (0.43–1.41)	<DL (<DL-0.97)	1.09 (0.19–2.04)	0.115
	≥0.9 μg/L	58.8	36.4	47.4	0.184	49.2	30.8	58.3	0.351
MCPP	≥DL, %	70.6	87.9	84.2	0.183	77.0	92.3	83.3	0.437
	μg/g-cre	0.55 (<DL-0.84)	0.30 (0.17–0.57)	0.25 (0.11–0.40)	0.223	0.34 (0.12–0.73)	0.35 (0.21–0.53)	0.31 (0.11–0.83)	0.993
	μg/L	0.72 (<DL-1.22)	0.35(0.26–0.63)	0.32 (0.26–0.70)	0.150	0.49 (0.24–1.14)	0.43 (0.29–0.65)	0.61 (0.29–1.30)	0.608
	≥0.5 μg/L	70.6^a^	36.4^b^	36.8^b^	0.009	49.2	46.2	58.3	0.808
MCiOP	μg/g-cre	1.8 (1.1–3.2)	2.0 (1.1–3.1)	1.4 (0.8–3.7)	0.776	1.8 (1.1–2.9)	2.0 (1.0–3.4)	1.7 (0.8–6.0)	0.953
	μg/L	2.5 (1.7–4.7)	2.5 (1.2–4.5)	2.4 (1.5–4.7)	0.860	2.4 (1.5–4.2)	2.9 (1.4–4.7)	3.2 (1.7–7.6)	0.667
MiNP	≥DL, %	14.7	9.1	10.5	0.752	14.8	0.0	8.3	0.299
MEHP	≥DL, %	100.0	97.0	94.7	0.449	98.4	100.0	91.7	0.310
	μg/g-cre	4.7 (2.4–8.0)	4.4 (2.4–8.6)	3.9 (2.5–6.5)	0.732	4.3 (2.4–8.2)	4.9 (3.1–6.3)	3.5 (2.5–6.6)	0.758
	μg/L	5.7 (3.8–11.2)	6.1 (4.0–8.8)	5.8 (2.9–9.8)	0.747	6.1 (3.6–9.3)	5.8 (4.1–7.5)	5.7 (3.1–11.5)	0.831
MEHHP	μg/g-cre	35.4 (22.2–53.2)	40.5 (22.4–68.2)	42.5 (20.4–53.0)	0.568	35.8 (21.4–52.4)	47.1 (31.4–64.6)	47.0 (22.5–58.5)	0.265
	μg/L	45.9 (31.0–71.1)	55.7 (31.8–90.5)	56.1 (28.0–92.6)	0.740	46.2 (28.4–72.4)	59.5 (32.6–99.3)	79.7 (32.2–101.5)	0.168
	≥50 μg/L	44.1	60.6	52.6	0.401	45.9	69.2	66.7	0.175
MECPP	μg/g-cre	9.6 (6.4–15.1)	11.2 (6.6–20.0)	11.4 (5.4–19.3)	0.582	9.9 (6.4–16.2)	12.0 (5.4–17.9)	15.5 (5.7–24.1)	0.650
	μg/L	14.9 (9.5–19.3)	17.6 (7.3_25.6)	12.1 (8.1–29.8)	0.905	15.0 (8.3–21.6)	16.3 (8.4–22.5)	25.5 (9.1–35.1)	0.287
MEOHP	μg/g-cre	8.0 (6.0–12.6)	8.2 (5.2–11.2)	9.3 (2.4–10.4)	0.712	7.8 (5.3–11.4)	9.3 (5.9–10.3)	9.9 (3.1–16.1)	0.808
	μg/L	11.3 (9.2–17.3)	10.7 (5.7–16.0)	7.6 (3.9–18.9)	0.334	10.6 (6.7–16.2)	10.7 (7.4–17.4)	12.7 (4.6–33.6)	0.759
MCMHP	μg/g-cre	4.5 (3.0–6.2)	4.1 (2.4–7.4)	3.6 (2.4–6.4)	0.526	4.4 (2.8–6.4)	4.0 (2.8–7.0)	4.7 (2.5–8.1)	0.919
	μg/L	6.3 (4.7–9.6)	6.2 (3.4–9.4)	5.5 (2.5–9.3)	0.513	6.0 (3.9–8.1)	5.7 (3.3–9.4)	8.8 (3.9–13.1)	0.491
ΣDEHP metabolites	μg/g-cre	69 (43–98)	69 (50–116)	77 (34–102)	0.758	69 (43–96)	92 (56–111)	83 (37–112)	0.509
	μg/L	87 (61–124)	102 (60–154)	90 (50–171)	0.817	89 (59–133)	102 (64–157)	138 (55–191)	0.336
Primary/secondary DEHP	ratio	8.1 (4.7–11.7)	6.7 (4.6–10.4)	6.7 (4.6–8.0)	0.472	7.6 (4.7–11.9)	6.7 (4.4–8.3)	5.2 (4.3–7.7)	0.118
	≥6.9	58.8	45.5	42.1	0.406	55.7	46.2	25.0	0.144
**Low molecular weight phthalate metabolites**									
MBzP	≥DL, %	5.9^a^	33.3^b^	42.1^b^	0.004	14.8^a^	46.2^b^	50.0^b^	0.005
MnBP	μg/g-cre	15.6 (11.5–21.9)	12.1 (7.7–21.7)	15.0 (8.4–25.3)	0.218	13.4 (10.0–21.2)	18.0 (11.2–26.7)	13.3 (7.5–31.2)	0.748
	μg/L	26.4 (18.7–32.8)^a^	15.1 (11.1–20.5)^b^	20.6 (15.7–36.4)^ab^	0.003	20.0 (13.9–29.2)	18.8 (15.0–35.3)	22.1 (5.9–48.4)	0.488
	≥20 μg/L, %	70.6^a^	27.3^b^	52.6^ab^	0.002	49.2	46.2	58.3	0.808
MiBP	μg/g-cre	15.6 (10.6–25.8)	13.2 (9.5–22.1)	16.8 (8.9–27.7)	0.733	14.9 (10.2–23.4)	14.1 (12.4–27.2)	15.6 (9.8–32.9)	0.777
	μg/L	25.4 (17.5–35.1)^a^	16.1 (14.1–21.6)^b^	20.9 (17.5–49.3)^ab^	0.012	19.9 (15.5–30.5)	21.0 (15.9–34.4)	25.5 (18.3–51.4)	0.190
	> 20 μg/L, %	70.6^a^	30.3^b^	57.9^ab^	0.004	49.2	61.5	58.3	0.651
ΣDBP metabolites	μg/g-cre	31 (23–47)	26 (18–43)	32 (18–53)	0.442	28 (20–46)	37 (24–52)	29 (17–64)	0.731
	μg/L	52 (36–66)^a^	31 (27–41)^b^	46 (35–84)^ab^	0.004	40 (29–59)	40 (31–65)	48 (35–99)	0.328
	≥40 μg/L, %	73.5^a^	30.3^b^	52.6^ab^	0.002	50.8	53.8	58.3	0:886
MEP	≥DL, %	97.1	93.9	94.7	0.824	95.1	92.3	100	0.648
	μg/g-cre	12.6 (5.5–40.9)	10.4 (3.2–31.5)	23.0 (6.1–85.8)	0.223	9.3 (4.3–31.5)	23.1 (10.5–114.1)	24.2 (6.4–80.1)	0.060
	μg/L	21.8 (9.0–43.5)	16.2 (3.9–38.6)	35.7 (12.5–123.6)	0.122	16.2 (6.3–37.9)^a^	27.2 (13.9–118.2)^ab^	44.2 (13.1–131.3)^b^	0.036
	≥20 μg/L, %	55.9	39.4	63.2	0.199	45.9	61.5	66.7	0.303
MMP	≥DL, %	88.2	87.9	94.7	0.702	86.9	100.0	91.7	0.362
	μg/g-cre	2.5 (1.4–4.8)	1.1 (0.3–4.2)	1.5 (0.5–6.1)	0.209	1.8 (0.5–4.8)	2.4 (1.1–4.7)	1.5 (0.6–7.8)	0.785
	μg/L	3.6 (1.7–6.9)	1.5 (0.4–5.4)	2.7 (1.1–8.4)	0.118	2.4 (1.0–6.4)	2.8 (1.0–6.8)	2.4 (1.2–11.5)	0.892
	≥2.6 μg/L, %	61.8	36.4	52.6	0.111	47.5	61.5	50.0	0.657

^ab^Values having different letter were statistically significant

Data are presented as median (Q1-Q3), %

*ABPM* ambulatory blood pressure monitoring, *BP* blood pressure, *BPA* bisphenol A, *cre* creatinine, *DL* detectable level, *HT* hypertension, *WCHT* white coat hypertension, *MnBP* mono-n-butyl phthalate, *MBzP* mono-benzyl phthalate, *MCiNP* monocarboxy isononyl phthalate, *MCiOP* mono carboxy isooctyl phthalate, *MCMHP* mono 2-carboxymethylhexyl phthalate, *MCPP* mono 3-carboxypropyl phthalate, *MECPP* mono 2-ethyl-5-carboxypentyl phthalate, *MEHHP* mono 2-ethyl 5-hydroxyhexyl phthalate, *MEHP* mono 2-ethylhexyl phthalic acid, *MEOHP* mono 2-ethyl-5-oxy-hexyl phthalate, *MEP* monoethyl phthalate, *MiBP* monoisobutyl phthalate, *MiNP* monoisononyl phthalate, *MMP* monomethyl phthalate, *ΣDBP metabolites* sum of dibutyl phthalate metabolites, *ΣDEHP metabolites* sum of di (2-ethylhexyl) phthalate metabolites

The percentage of adolescents with urinary MBzP above detectable level was higher in ABPM-HT (42.1%) and WCHT groups (33.3%), compared to the normal blood pressure profile group (5.9%, *p* = 0.004).

The percentage of adolescents having ΣDBP metabolites > 40 μg/L, was lower in WCHT groups (30.3%), compared to the normal blood pressure profile group (73.5%, *p* = 0.004).

Multinominal logistic regression revealed that high MCPP, ΣDBP, and MMP metabolites showed negative association with WCHT, whereas, high MEHHP and detectable MBzP had positive association with WCHT following adjustment for age, sex, BMI-SDS, WBC, chloride, ACCa, and phosphate levels (Model 2). When these five metabolites (MCPP, ΣDBP metabolites, MEHHP, MBzP, and MMP) were included in analysis, this correlation for WCHT persisted only for detectable MBzP (OR 33.5; 95% CI 2.1–539.6), ΣDBP metabolites (OR 0.06; 95% CI 0.01–0.38), and MMP (OR 0.17; 95% CI 0.03–0.86). MCPP and MEHHP did not remain in the final model. There was a negative association between high MCPP and ABPM-HT, whereas, positive association between detectable MBzP and ABPM-HT in model 2 and model 3 (Table 3).

**Table 3:** Odds ratios for white coat hypertension (WCHT) and hypertension diagnosed by ambulatory blood pressure monitoring (ABPM hypertension) compared to normal blood pressure profile, comparing detectable or median levels of urinary phthalate metabolites

	WCHT vs. Normal BP profile				ABPM hypertension vs. Normal BP profile			
	OR (95% CI)	AOR (95% CI)^a^	AOR (95% CI)^b^	AOR (95% CI)^c^	OR (95% CI)	AOR (95% CI)^a^	AOR (95% CI)^b^	AOR (95% CI)^c^
MCiNP ≥0.9 μg/L	0.40 (0.15–1.07)	0.41 (0.15–1.14)	0.48 (0.14–1.65)		0.63 (0.20–1.95)	0.60 (0.18–2.05)	0.45 (0.09–2.30)	
MCPP ≥0.5 μg/L	**0.24 (0.09–0.66)**	**0.27 (0.09–0.76)**	**0.25 (0.08–0.87)**	0.28 (0.06–1.46)	0.24 (0.07–0.80)	0.28 (0.08–0.97)	**0.16 (0.03–0.91)**	**0.06 (0.01–0.74)**
MEHHP ≥50 μg/L	1.9 (0.7–5.2)	2.2 (0.8–6.1)	**3.5 (1.0–12.3)**	3.5 (0.5–23.9)	1.4 (0.5–4.3)	1.7 (0.5–5.8)	3.2 (0.6–16.6)	2.7 (0.3–24.3)
Primary/secondary DEHP ≥6.9 μg/L	0.58 (0.22–1.54)	0.70 (0.25–198)	0.92 (0.27–3.09)		0.51 (0.16–1.59)	0.72 (0.21–2.52)	0.95 (0.20–4.53)	
MBzP ≥DL	**8.0 (1.6–39.7)**	**8.5 (1.6–44.4)**	**6.0 (1.0–36.8)**	**33.5 (2.1–539.6)**	11.6 (2.1–63.3)	14.1 (2.3–86.8)	**15.2 (1.9–122.7)**	**50.5 (3.1–846.7)**
ΣDBP met≥40 μg/L	**0.16 (0.05–0.45)**	**0.16 (0.05–0.49)**	**0.12 (0.03–0.46)**	**0.06 (0.01–0.38)**	0.40 (0.12–1.30)	0.49 (0.14–1.75)	0.60 (0.11–3.22)	0.98 (0.09–10.12)
MEP ≥20 μg/L	0.51 (0.19–1.36)	0.64 (0.23–1.84)	0.71 (0.22–2.28)		1.35 (0.43–4.28)	1.85 (0.52–6.63)	2.80 (0.57–13.92)	
MMP ≥2.6 μg/L	**0.35 (0.13–0.95)**	**0.27 (0.09–0.79)**	**0.20 (0.05–0.71)**	**0.17 (0.03–0.86)**	0.69 (0.22–2.14)	0.49 (0.14–1.71)	0.35 (0.07–1.79)	0.15 (0.02–1.19)

Multinominal logistic regression analyzed the differences in rates of detectable MBzP among groups of some BP profiles after confounding factors

^a^ adjusted for age, sex, BMI-SDS

^b^ adjusted for age, sex, BMI-SDS, white blood cell, platelet, chloride, albumin-corrected calcium x phosphate product, eGFR, urinary creatinine

^c^ adjusted for age, sex, BMI-SDS, white blood cell, platelet, chloride, albumin-corrected calcium x phosphate product, eGFR, urinary creatinine, selected five phthalate metabolites

*BMI-SDS* body mass index standard deviation score, *DL* detectable level, *eGFR* estimated glomerular filtration rate, *MBzP* mono-benzyl phthalate, *MCiNP* monocarboxy isononyl phthalate, *MCPP* mono 3-carboxypropyl phthalate, *MEHHP* mono 2-ethyl 5-hydroxyhexyl phthalate, *MEP* monoethyl phthalate, *MMP* monomethyl phthalate

*ΣDBP metabolites* sum of dibutyl phthalate metabolites

Overall, 61 adolescents (70.9%) had normal daytime and night-time blood pressure, 13 (15.1%) had daytime or night-time HT and 12 (14.0%) had sustained HT (i.e. daytime and night-time HT). Adolescents with sustained HT had higher BMI-SDS and counts of platelets, compared to those with normal daytime and night-time blood pressure (*p* = 0.005 and *p* = 0.036, respectively; Table 1).

The percentage of adolescents with urinary MBzP equal to or greater than detectable level was higher in sustained HT (50.0%) and daytime or night time HT group (46.2%), compared to normal daytime and night-time blood pressure group (14.8%, *p* = 0.005, Table 2).

**Fig. 2:**
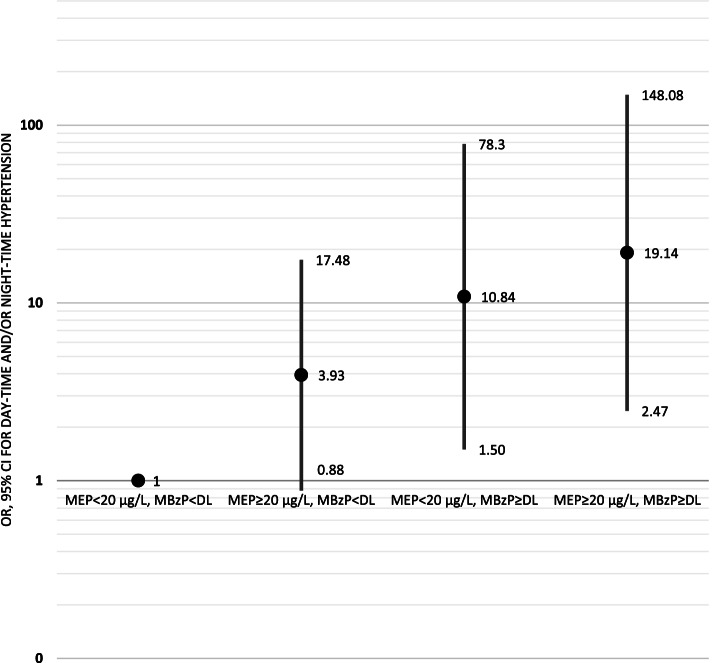
Odds ratios for day-time and/or night-time HT diagnosed by ABPM compared to “normal day-time and night-time blood pressure”, according to urinary MEP and MBzP levels

Urinary MBzP equal to or greater than detectable level was a risk factor for daytime and/or night-time HT (OR 5.3; 95% CI 1.8–15.3), compared to normal daytime and night-time blood pressure (Supplementary Table 3). This correlation persisted in all models (Supplementary Table 3). Following adjustment for age, sex, BMI-SDS, parental hypertension, platelet. eGFR, urinary creatinine, and selected three urinary phthalate metabolites (MEHHP, MBzP, MEP) adolescents having high MEP levels (≥20 μg/L) had increased Odds for “day-time and/or night-time HT” (OR 3.37, 95% CI 0.98–11.64), compared to “normal day-time and night-time blood pressure” (Supplementary Table 3). In a model combining MBzP and MEP groups; compared to low MEP with undetectable MBzP levels, adolescents having high MEP and detectable MBzP levels (OR 19.14) and those having low MEP and detectable MBzP levels (OR 10.84) had the highest risks for “day-time and/or night-time HT” (Fig. 2).

Urinary MBzP was equal to or greater than detectable level in 21 adolescents; adolescents in this group had higher percentage of parental HT (42.9% vs. 13.8%, *p* = 0.011), ACCa to phosphate ratio (2.3 ± 0.4 vs. 2.1 ± 0.3, *p* = 0.048), and lower serum phosphate (4.2 ± 0.6 vs. 4.5 ± 0.6, *p* = 0.039) and ACCa x phosphate product (38.6 ± 6.0 vs. 41.8 ± 5.9, *p* = 0.037), compared to group with urinary MBzP below detectable level (Table 4). The differences for other clinical parameters, complete blood count, and serum and urine biochemistry did not reach to statistical significance.

**Table 4:** Clinical characteristics and laboratory findings of adolescents according to urinary mono-benzyl phthalate (MBzP) level

Parameters	MBzP groups		P
	< Detectable level (*N* = 65)	≥ Detectable level (*N* = 21)	
Age (years)	13.2 ± 0.7	13.5 ± 0.9	0.097
Female/male, % / %	53.8/46.2	38.1/61.9	0.209
Height-SDS	0.5 ± 1.0	0.7 ± 0.8	0.368
BMI-SDS	0.8 ± 1.2	1.2 ± 1.2	0.266
Obesity, %	20.0	28.6	0.545
Parental hypertension, %	13.8	42.9	0.011
Carotid IMT-SDS	0.51 ± 0.50	0.48 ± 0.51	0.805
Hemoglobin (g/dl)	14.0 ± 0.9	14.5 ± 1.2	0.070
White blood cell (×10^3^/mm^3^)	7.7 ± 2.4	7.7 ± 1.6	0.947
Platelets (×10^3^/mm^3^)	277 ± 63	289 ± 54	0.448
Serum protein (g/dl)	7.6 ± 0.4	7.7 ± 0.4	0.366
Serum albumin (g/dl)	4.6 ± 0.3	4.8 ± 0.3	0.046
Serum globulin (g/dl)	3.0 ± 0.3	2.9 ± 0.3	0.373
Blood urea nitrogen (mg/dl)	11.1 ± 2.8	11.4 ± 2.8	0.721
Creatinine (mg/dl)	0.6 ± 0.1	0.6 ± 0.1	0.124
Uric acid (mg/dl)	5.0 ± 1.2	5.4 ± 1.1	0.208
Sodium (mEq/L)	139.0 ± 1.5	138.5 ± 1.5	0.164
Potassium (mEq/L)	4.2 ± 0.3	4.3 ± 0.3	0.607
Chloride (mEq/L)	104.4 ± 1.9	103.7 ± 2.3	0.186
Calcium (mg/dl)	9.8 ± 0.4	9.9 ± 0.4	0.543
ACCa (mg/dl)	9.3 ± 0.3	9.3 ± 0.3	0.453
Phosphate (mg/dl)	4.5 ± 0.6	4.2 ± 0.6	0.049
ACCa-phosphate product (mg^2^/dl^2^)	41.8 ± 5.9	38.6 ± 6.0	0.037
ACCa-phosphate ratio (mg/mg)	2.10 (1.91–2.35)	2.15 (1.96–2.56)	0.118
Magnesium (mg/dl)	2.04 ± 0.14 (*N* = 51)	2.05 ± 0.15 (*N* = 16)	0.731
Urinary creatinine (mg/dl)	154.8 ± 65.2	161.5 ± 81.6	0.705
UPCR (mg/mg)	0.08 (0.07–0.11)	0.09 (0.07–0.12)	0.633
UACR (mg/g)	6.7 (4.3–15.3)	7.5 (3.9–20.4)	0.608
UBCR (μg/g)	47.6 (22.9–71.8)	63.9 (24.5–106.9)	0.181

Data are presented as % or mean ± standard deviation or median (Q1-Q3) as appropriate

*ACCa* albumin-corrected calcium, *BMI* body mass index, *IMT* intima media thickness, *SDS* standard deviation score, *UACR* urinary albumin to creatinine ratio, *UBCR* urinary beta-2 microglobulin to creatinine ratio, *UPCR* urinary protein to creatinine ratio

Adolescents with urinary MBzP equal to or greater than detectable level had higher 24-h MAP-SDS, 24-h systolic blood pressure-SDS, daytime systolic blood pressure-SDS, night-time MAP-SDS, night-time systolic blood pressure-SDS, and night-time diastolic blood pressure-SDS (Table 5), compared to counterparts.

**Table 5:** Comparison of ambulatory blood pressure monitoring parameters and carotid intima media thickness according to urinary mono-benzyl phthalate (MBzP) level

Parameters	MBzP groups		p
	< Detectable level (*N* = 65)	≥ Detectable level (*N* = 21)	
24-h MAP-SDS	0.7 ± 0.9	1.2 ± 1.0	0.021
≥95 percentile, %	16.9	38.1	0.067
24-h Systolic BP-SDS	0.5 ± 1.1	1.2 ± 1.0	0.008
≥95 percentile, %	13.8	23.8	0.315
24-h Diastolic BP-SDS	0.2 ± 0.8	0.5 ± 1.1	0.149
≥95 percentile, %	4.6	19.0	0.057
24-h Systolic and/or Diastolic BP ≥95 percentile, %	16.9	33.3	0.129
Daytime MAP-SDS	0.5 ± 0.9	1.0 ± 1.1	0.056
≥95 percentile, %	12.3	33.3	0.044
Daytime Systolic BP-SDS	0.4 ± 1.0	1.1 ± 1.1	0.010
≥95 percentile, %	12.3	23.8	0.291
Daytime Diastolic BP-SDS	0.0 ± 0.9	0.3 ± 1.3	0.286
≥95 percentile, %	4.6	23.8	0.019
Daytime Systolic and/or Diastolic BP ≥95 percentile, %	15.4	38.1	0.035
Night-time MAP-SDS	0.8 ± 0.8	1.5 ± 0.9	0.002
≥95 percentile, %	10.8	52.4	< 0.001
Night-time Systolic BP-SDS	0.6 ± 0.9	1.4 ± 0.9	0.001
≥95 percentile, %	12.3	42.9	0.004
Night-time Diastolic BP-SDS	0.4 ± 0.7	0.9 ± 0.8	0.008
≥95 percentile, %	3.1	23.9	0.009
Night-time Systolic and/or Diastolic BP ≥95 percentile, %	13.8	47.6	0.002
Non-dippers			
Systolic BP non-dipper, %	33.8	42.9	0.455
Diastolic BP non-dipper, %	1.5	9.5	0.146
MAP non-dipper, %	16.9	14.3	0.776
24-h heart rate SDS	0.5 ± 0.9	0.5 ± 1.0	0.963
≥95 percentile, %	9.2	9.5	1.000
Daytime heart rate SDS	0.4 ± 0.9	0.4 ± 1.0	0.876
≥95 percentile, %	6.2	4.8	1.000
Night-time heart rate SDS	0.6 ± 0.9	0.5 ± 1.0	0.671
≥95 percentile, %	12.3	14.3	1.000
cIMT-SDS	1.3 ± 1.2 (*N* = 64)	1.5 ± 1.1 (*N* = 19)	0.395
≥95 percentile, %	37.2	42.1	0.717

Data are presented as % or mean ± standard deviation as appropriate

*BP* blood pressure, *cIMT* carotid intima media thickness, *MAP* mean arterial pressure, *SDS* standard deviation score

From 83 adolescents, cIMT was measured, cIMT-SDS was equal to or greater than the 95th percentile in 32 adolescents (38.6%). There was no association between clinical characteristics and cIMT (Table 6). Adolescents having cIMT-SDS ≥95th percentile showed higher frequencies of ΣDEHP metabolites ≥100 μg/L compared to the adolescents with normal cIMT (Table 7). The correlation was not changed following adjustment for age, sex, BMI-SDS, eGFR and U-cre (OR 3.05; %95 CI 1.16–8.02, Supplementary Table 4).

**Table 6:** Comparison of clinical characteristics according to carotid intima media thickness groups

Parameters	cIMT groups		p
	< 95 percentile (*N* = 51)	≥95 percentile (*N* = 32)	
Female/male, % / %	54.9/45.1	46.9/53.1	0.476
Age (years)	13.3 ± 0.7	13.3 ± 0.6	0.656
Height-SDS	0.52 ± 0.95	0.71 ± 0.83	0.345
BMI-SDS	0.78 ± 1.14	1.06 ± 1.17	0.292
Obesity, %	15.7	28.1	0.172
Parental characteristics			
Mother’s age (years)	40.8 ± 6.0	40.8 ± 6.1	0.981
Father’s age (years)	44.7 ± 6.6	45.8 ± 7.2	0.496
Parental hypertension, %	17.6	21.9	0.635
Laboratory findings			
Hemoglobin (g/dl)	14.2 ± 0.9	14.1 ± 1.2	0.659
White blood cell (×10^3^/mm^3^)	7.6 ± 2.2	8.0 ± 2.3	0.440
Platelets (×10^3^/mm^3^)	279 ± 55	282 ± 73	0.792
Serum protein (g/dl)	7.7 ± 0.4	7.6 ± 0.4	0.609
Serum albumin (g/dl)	4.7 ± 0.3	4.6 ± 0.3	0.544
Serum globulin (g/dl)	3.0 ± 0.3	2.9 ± 0.3	0.859
Blood urea nitrogen (mg/dl)	11.1 ± 2.5	11.6 ± 3.3	0.411
Creatinine (mg/dl)	0.57 ± 0.09	0.57 ± 0.12	0.873
eGFR [Schwartz] (ml/min/1.73m^2^)	119.5 ± 18.2	122.7 ± 21.7	0.480
Uric acid (mg/dl)	5.1 ± 1.1	5.2 ± 1.1	0.592
Sodium (mEq/L)	139.0 ± 1.3	138.7 ± 1.8	0.349
Potassium (mEq/L)	4.3 ± 0.3	4.1 ± 0.3	0.072
Chloride (mEq/L)	104.2 ± 2.1	104.2 ± 1.9	0.864
Calcium (mg/dl)	9.9 ± 0.4	9.8 ± 0.3	0.309
ACCa (mg/dl)	9.3 ± 0.3	9.3 ± 0.3	0.387
Phosphate (mg/dl)	4.4 ± 0.6	4.4 ± 0.6	0.952
ACCa-phosphate product (mg^2^/dl^2^)	40.9 ± 6.0	40.7 ± 5.7	0.896
ACCa-phosphate ratio (mg/mg)	2.12 (1.93–2.40)	2.13 (1.93–2.35)	0.837
Magnesium (mg/dl)	2.06 ± 0.14	2.03 ± 0.15	0.388
UPCR (mg/mg)	0.08 (0.07–0.11)	0.08 (0.07–0.12)	0.489
UACR (mg/g)	7.4 (4.0–16.9)	6.5 (4.5–19.5)	0.842
UBCR (μg/g)	56.3 (23.0–74.1)	54.4 (20.2–71.3)	0.955

Data are presented as mean ± standard deviation, median (Q1-Q3) or %

*ACCa* albumin-corrected calcium, *BMI* body mass index, *cIMT* carotid intima media thickness, *eGFR* estimated glomerular filtration rate, *SDS* standard deviation score, *UACR* urinary albumin to creatinine ratio, *UBCR* urinary beta-2 microglobulin to creatinine ratio, *UPCR* urinary protein to creatinine ratio

**Table 7:** Comparison of urinary bisphenol A (BPA) and phthalate metabolites according to according to carotid intima media thickness groups

Parameters		cIMT groups		p
		< 95 percentile (*N* = 51)	≥ 95 percentile (*N* = 32)	
Urinary BPA				
Total BPA	μg/g-cre	88 (53–149)	96 (53–174)	0.896
	μg/L	142 (94–195)	127 (84–170)	0.407
Free BPA	≥DL, %	15.7	9.4	0.517
**High molecular weight phthalate metabolites**				
MCiNP	μg/g-cre	0.57 (0.10–0.84)	0.71 (0.27–1.10)	0.251
	μg/L	0.76 (0.00–1.43)	0.87 (0.42–1.24)	0.605
	≥DL,%	74.5	81.3	0.477
	≥0.9 μg/L, %	45.1	46.9	0.874
MCPP	μg/g-cre	0.34 (0.10–0.64)	0.33 (0.14–0.71)	0.792
	μg/L	0.59 (0.24–1.12)	0.34 (0.26–0.78)	0.676
	≥DL,%	76.5	84.4	0.385
	≥0.5 μg/L, %	56.9	37.5	0.086
MCiOP	μg/g-cre	1.47 (0.97–2.57)	1.99 (1.31–3.57)	0.169
	μg/L	2.3 (1.5–4.0)	2.5 (1.6–5.0)	0.466
MiNP	≥DL,%	7.8	15.6	0.297
MEHP	μg/g-cre	3.4 (2.2–6.5)	5.2 (3.2–8.2)	0.083
	μg/L	5.7 (3.0–8.6)	6.4 (4.7–9.8)	0.147
	≥5.8 μg/L, %	45.1	53.1	0.476
MEHHP	μg/g-cre	34.8 (17.8–50.1)	42.2 (28.8–62.0)	0.040
	μg/L	45.4 (29.1–70.5)	62.0 (33.5–93.8)	0112
	≥50 μg/L, %	45.1	59.4	0.205
MECPP	μg/g-cre	7.9 (5.2–14.9)	13.9 (8.6–21.2)	0.007
	μg/L	13.6 (7.8–19.7)	17.9 (9.4–27.7)	0.067
	≥15 μg/L, %	45.1	62.5	0.123
MEOHP	μg/g-cre	8.0 (4.7–10.7)	8.4 (5.4–11.7)	0.519
	μg/L	10.7 (6.6–16.3)	10.7 (6.6–18.4)	0.963
MCMHP	μg/g-cre	3.6 (2.5–6.4)	4.5 (3.2–6.5)	0.227
	μg/L	5.6 (3.5–8.4)	6.5 (3.8–10.5)	0.507
ΣDEHP metabolites	μg/g-cre	60.2 (37.6–91.7)	76.7 (50.0–114.9)	0.046
	μg/L	87.3 (56.6–127.8)	117.5 (60.8–160.7)	0.123
	≥100 μg/L, %	35.3	59.4	0.032
Primary/secondary DEHP metabolites	Ratio	6.5 (4.5–9.0)	8.2 (4.7–11.6)	0.207
**Low molecular weight phthalate metabolites**				
MBzP	≥DL, %	21.6	25.0	0.717
MnBP	μg/g-cre	13.3 (10.4–20.6)	13.5 (9.1–28.3)	0.708
	μg/L	20.0 (14.7–28.2)	20.7 (13.6–32.4)	0.978
MiBP	μg/g-cre	14.0 (10.0–22.4)	15.9 (10.3–33.0)	0.317
	μg/L	21.0 (15.6–26.4)	19.1 (15.6–36.3)	0.581
ΣDBP metabolites	μg/g-cre	26.7 (20.2–43.3)	29.0 (19.0–61.3)	0.531
	μg/L	40.3 (30.8–53.7)	38.3 (29.4–69.1)	0.779
MEP	μg/g-cre	11.8 (5.2–24.7)	19.4 (5.3–67.9)	0.340
	μg/L	18.3(9.8–34.3)	31.4 (8.0–82.4)	0.416
MMP	≥20 μg/L, %	49.0	53.1	0.716
	μg/g-cre	1.88 (0.56–4.18)	1.60 (0.48–6.02)	0.903
	μg/L	2.67 (1.07–6.33)	2.02 (0.82–5.19)	0.613

Data are presented as % or median (Q1-Q3)

*cre* creatinine, *MnBP* mono-n-butyl phthalate, *MBzP* mono-benzyl phthalate, *MCiNP* monocarboxy isononyl phthalate, *MCiOP* mono carboxy isooctyl phthalate, *MCMHP* mono 2-carboxymethylhexyl phthalate, *MCPP* mono 3-carboxypropyl phthalate, *MECPP* mono 2-ethyl-5-carboxypentyl phthalate, *MEHHP* mono 2-ethyl 5-hydroxyhexyl phthalate, *MEHP* mono 2-ethylhexyl phthalic acid, *MEOHP* mono 2-ethyl-5-oxy-hexyl phthalate, *MEP* monoethyl phthalate, *MiBP* monoisobutyl phthalate, *MiNP* monoisononyl phthalate, *MMP* monomethyl phthalate, *ΣDBP metabolites* sum of dibutyl phthalate metabolites, *ΣDEHP metabolites* sum of di (2-ethylhexyl) phthalate metabolites

## Discussion

Our study indicates differences for BMI-SDS, WBC, platelet count, serum calcium, ACCa, phosphate levels, ACCa-phosphate product, and ACCa to phosphate ratio when the adolescents were grouped according to blood pressure profile groups. Moreover, urinary MBzP above detectable level was identified as an independent risk factor for ABPM-HT and WCHT following adjustment for age, sex, BMI-SDS, WBC, chloride, ACCa, and phosphate levels; and for daytime or night-time HT and sustained HT following adjustment for age, sex, and BMI-SDS.

### Serum electrolytes and blood pressure

Early studies showed that chloride, rather than sodium, may be crucial for HT [13]. In a study by Kurtz and Morris, salt-sensitive HT was induced by a high NaCl diet; but a non-chloride diet with similar Na loading failed to induce HT [47]. Wilcox had previously shown that hyperchloremia induced renal vasoconstriction [13]. In contrast to these findings, a recent study showed a J-shaped association of chloride with mortality and cardiovascular events; the lowest chloride quartile (≤103.9 mEq/L) had significantly higher all-cause mortality in a group of pre-dialysis patients (median chloride was 106.0 mEq/L) [10]. In parallel to their finding, in our study, serum chloride level was lower in WCHT (103.8 ± 2.0) and ABPM-HT group (103.5 ± 2.2), compared to normal blood pressure group (105.0 ± 1.8). Several mechanisms may have a role in the association between low chloride and HT: a) a decrease in NaCl concentration in the macula densa of the kidneys increases renin secretion and results in the activation of the renin-angiotensin system and retention of sodium and water [48]; b) low chloride may cause inflammation, the highest CRP level was reported in these cases [10]. Additionally, similar to the previous studies [49–52], our results revealed associations between high blood pressure and elevated WBC count and platelet count even after controlling BMI-SDS. Platelets are known to have important inflammatory functions [53]. Considering that HT is an inflammatory process [6], it was not unexpected that both WBC and platelet counts increased in hypertensive patients compared to those with normal blood pressure. In parallel with our finding, a study conducted with children showed that WBC count was higher in hypertensive patients, regardless of dipping status [50]. In two other studies in adults, it was stated that the WBC count was higher in hypertensive patients and could be used as a risk factor for HT [51, 52].

Serum calcium and ACCa were both higher in the ABPM-HT group, compared to WCHT. Although it did not reach to statistical significance, serum phosphate was lowest in the ABPM-HT group; and lower in WCHT, compared to normal blood pressure group. This trend reflected as higher ACCa to phosphate ratio in the ABPM-HT group and lower ACCa-phosphate product in WCHT, compared to normal blood pressure group. Previous studies have revealed different results for a correlation between serum calcium and HT. In a study by Hazari et al., where the variables were not adjusted by age, BMI, total cholesterol, triglycerides, serum calcium had no effect on HT [54]. But, cross-sectional studies in adult populations from Norway [55] and United States [56], and a recent longitudinal study from Taiwan [8] indicated a positive correlation between serum calcium and HT. The study from Taiwan showed also the association of higher serum calcium levels with metabolic syndrome and diabetes [8]. Calcium may lead to the development of HT by different mechanisms: a) influx of calcium into the smooth muscle of the artery leading to muscle contracture and increase in vascular resistance, b) positive correlation between calcium and cholesterol [55], c) correlation between calcium and PTH may lead to the production of collagen by aortic vascular smooth muscle cells and thickening of vascular wall [57]. Low phosphate, on the other hand, is related to HT, metabolic syndrome, and increased sympathoadrenal activity [11, 12]. Serum phosphate was found to be inversely related to blood pressure in normotensive individuals and to be lowest in hypertensive patients [9, 58]. In a study conducted by Vyssoulis, among 2600 adult patients with WCHT, decreased levels of serum phosphate and calcium-phosphate product were associated with a higher incidence of a non-dipping nocturnal systolic blood pressure and an impaired metabolic profile [59]. In patients with mild essential HT, low phosphate is inversely related to sympathetic adrenal tone and may be caused by increased plasma epinephrine within pathophysiologic arterial concentrations [60]. Epinephrine leads to a shift of phosphate from the extracellular to the intracellular compartment [12]. We cannot conclude, however, a definite cause-and-effect relationship between low phosphate and HT with our study design. Since the relationship between HT and low levels of serum phosphate may be associated with an unbalanced diet (low phosphate and high carbohydrate consumption) [12].

### Phthalate metabolites and blood pressure

The percentage of detection of MBzP, one of the phthalate metabolites, in the urine was significantly higher in both the WCHT group and the ABPM-HT group compared to the normal blood pressure group. In addition, detectable MBzP remained an independent predictor of either WCHT or ABPM-hypertension. Studies evaluating the relationship between urinary phthalates and blood pressure in children and adolescents are limited and reported controversial results. While an increased risk for blood pressure was found in some studies with urinary MBzP levels in children [61] and adults [62], no correlation was observed in others [63, 64]. In a cross-sectional study of 108 children aged 6–18 years, a positive correlation was reported between urinary MBzP level and systolic blood pressure [61].

In our study, the frequencies of MEP above or equal to median levels were slightly higher in daytime and/or night-time HT group compared to normal blood pressure group. MEP showed an additive interaction with MBzP on daytime and/or night-time HT. However, a study from China in 276 children aged 6–8 years, evaluating 11 phthalate metabolites (MnBP, MEP, MMP, MBzP, MCOP, MCPP, MOP, MEHP, MECPP, MEHHP, and MEOHP) in urine, in boys a 1-ng/ml revealed that an increase in MEP concentration was associated with a 0.016 mmHg decrease in systolic blood pressure [63].

Except for urinary MBzP and MEP levels, other phthalate metabolites showed no significant positive association with blood pressure profiles in our study. This is the first study evaluating WCHT with these pollutants. A recent cross-sectional study from Isfahan (an industrial city in Iran) included 108 children (6–18 years) observed a positive relationship between systolic blood pressure and some metabolites including urinary MnBP and MEHP; no association with MEHHP, MEOHP, and MMP [61]. A recent cross-sectional study from China including a total of 1044 primary school children (6–8 years old) with an electronic sphygmomanometer were studied reported that MMP, MiBP, MnBP, MCEPP, MCMHP, “the sum of MMP, MEP, MiBP, and MnBP”, and “the sum of MCEPP, MEHHP, MEOHP, MCMHP, MEHP, MMP, MEP, MiBP, and MnBP” in urine samples were associated with elevations in systolic/diastolic blood pressure-SDS, pulse pressure, and MAP. Urine MMP level was also significantly associated with the risk of high blood pressure (blood pressure ≥ the 90th percentile for sex/age/height) [65]. National Health and Nutrition Examination Survey (NHANES 2001–2010; aged 20–80 years) showed relationships between high blood pressure and MECPP, MnBP, MEHHP, MMP, MEOHP, and MBzP in the adult age group after adjusting for urinary creatinine, age, sex, ethnicity, and body mass index [62]. The relationship between urinary phthalates and blood pressure (using an aneroid sphygmomanometer) was examined in cross-sectional analyses using a subsample of US children and adolescents (8–19 years) in 2009 to 2012 NHANES. Di-2-ethylhexylphthalate, di-isononyl phthalate, and di-isodecyl phthalate were associated with higher blood pressure (age-, sex- and height-standardized). No association was detected between LMW phthalates and blood pressure [66]. In the Dutch general population (662 adults) blood pressure was not associated with any of the urinary MEP, MiBP, MnBP, MEHHP, MEOHP, MECPP, MBzP, MEHP, and MMP. On the other hand, they reported nonlinear significant associations for MiBP quartiles with systolic blood pressure compared to the first quartile, lowest exposure [67]. The sum of LMW phthalate metabolites and the sum of HMW phthalate metabolites with the analyses of serially assessed exposure (6 samples per case) were not found to be associated with blood pressure in a cohort of 538 children with chronic kidney disease [68]. A population-based, prospective cohort study among 1064 mother-child pairs revealed sex-specific differences for phthalates on blood pressure; higher third-trimester maternal urine concentrations of HMW phthalates, di-2-ehtylhexylphthalate, and di-n-octylphthalate were associated with lower systolic and diastolic blood pressure among girls [69].

In our study a negative association was detected between ΣDBP metabolites and WCHT. In a mouse model, Xie et al. showed an increase in the levels of angiotensin-converting enzyme (ACE) and angiotensin II (AngII) in the DEHP treatment group without a significant change in estradiol level; on the other hand, there was an increase in the level of estradiol in the DBP treatment group, and the expression of ACE, AngII, AT1R, and eNOS in the DBP treatment groups showed no significant change. They suggested that different effects of DEHP and DBP on blood pressure could be related with the different estradiol levels induced with DEHP and DBP [70].

We showed a negative association between MMP and WCHT. In a study conducted by Yao et al. in children, a positive relationship was reported between MMP and high blood pressure [65]. In addition, an inverted-U-shaped relationship with atherosclerosis, which is one of the cardiovascular risk factors, has been reported in adults [71]. This suggests that different effects can be seen at different doses rather than a linear dose-response relationship. A negative association between insulin resistance and MMP in children was reported by Hashemi et al. [61]. It has been reported that insulin resistance and compensatory hyperinsulinemia may lead to increased blood pressure by causing sympathetic system activation, vascular changes, insufficient vascular dilatation and changes in membrane ion exchange [72–74]. These may be the underlying reasons for this negative relationship we have found.

In our study, we also showed a negative relation between MCPP and ABPM hypertension. Although higher MCCP concentrations in the first-trimester was found to be associated with pregnancy induced hypertension [75], MCPP was has not been found to be associated with hypertension. Our literature search did not reveal any explanation. Further studies are needed to explain these discrepancies.

The results of the studies are inconsistent, the number and type of metabolites studied are not standardized. Differences might also be due to sample matrix, age groups, exposed dosage, and whether the metabolite level is used directly or corrected by urinary creatinine or urine density. In addition, exposure to multiple contaminants is present at the same time and this might cause interaction between pollutants.

In our study, there is no interaction between carotid IMT-SDS and MBzP. We have also shown that phosphate was significantly lower in adolescents with urinary MBzP equal to or greater than the detectable level. Interestingly, a previous study detected both damage in liver and kidney and abnormalities in the trace element and mineral levels DEHP-administered rats [28]. Therefore, additional studies are needed to evaluate the associations of phthalates with blood pressure in different micronutrient status.

Carotid IMT-SDS was found to be associated with ΣDEHP metabolites in our study. Similarly, matrix metalloproteinases-2 and -9 expression which are inducers of atherosclerosis was reported to be increased in rats exposed to DEHP compared with control rats [76]. In addition, MEHP, ΣDEHP, and MnBP exposures in a human study are strongly found to be associated with thicker CIMT in adolescents and young adults in Taiwan [77].

Although it is not known exactly how phthalates cause changes in blood pressure, it is thought that oxidative damage may be responsible. Interestingly, a positive association between MBzP and increased oxidative stress and impaired vascular function was reported in the pediatric age group [78]. There are some proposed mechanisms in experimental models. In a mice study, exposure to DINP, another HMW phthalate, was shown to increase systolic blood pressure, diastolic blood pressure, and MAP, decrease endothelial nitric oxide synthase expression, and nitric oxide production [79]. Exposure to DEHP was found to cause an increase in mouse blood pressure through the renin-angiotensin-aldosterone system depending on different estradiol levels [70].

### BPA and blood pressure

In our study, we did not find a significant relationship between the tBPA levels and blood pressure profiles. Similarly, a study conducted with 471 Dutch children aged 6–10 years reported that there was no significant relationship between blood pressure and BPA after multiple testing corrections [27]. However, in a study conducted with 132 children aged 6–18 years in Iran, the urinary BPA concentrations of the participants were found to be 282.53 ± 166.02 μg/g-cre and a linear increase in blood pressure was reported among the tertiles determined according to the BPA concentration [80]. In a study examining 39 obese and overweight children aged 3–8 years, a positive correlation was reported between urinary BPA levels and diastolic blood pressure in girls. No relationship was found between BPA and diastolic blood pressure in boys and between BPA and systolic blood pressure in both genders [81]. A multi-center prospective cohort study of children aged 6 months to 16 years with mild-to-moderate chronic kidney disease showed no interaction between blood pressure and urinary BPA levels [68]. No association was found between blood pressure and urinary BPA levels in 662 native Dutch adult subjects [67]. Analytic method, exposed dosage, obesity, and age groups of children might influence the results. We detected high BPA levels in our study. However, the frequency of fBPA above the detection limit was more in WCHT and APBM-HT groups compared to the normal blood pressure profile groups (*p* = 0.081). Although there was no significant association, further studies are needed with a larger sample size. fBPA is considered to be more toxicologically active than the conjugated BPA [82]. There are no studies evaluating the interaction between fBPA and cardiovascular events.

We identified a higher percentage in parental HT in adolescents with HT. Similarly, familial aggregation with an increased liability of childhood-onset essential HT with parental essential HT is known [83]. Besides genetic predisposition, the same environmental exposures might have a role in theses aggregation.

### Strengths and limitations

In our study, we focused on asymptomatic adolescents detected in school screening, without antihypertensive medication. Patients with heart disease and having any drug therapy were excluded to avoid bias. In patients, different treatment interventions may affect the exposure level. The inclusion of asymptomatic adolescents without hypertensive medication might influenced the results and only statistically significant association could be detected with the phthalate MBzP. The small sample size might also affect the detection of associations. The cross-sectional design, relatively small study group, lack of detailed data for exposure, a single measurement of urinary metabolites were the limitations of the study. To some extent, these features might limit the generalizability of the current findings. Fetal origins of HT could not be evaluated in our study due to the single-exposure design. However, prenatal pollutant exposure might have an additive role in childhood blood pressure [64]. To the best of our knowledge, for the first time, 24-h ABPM was performed to examine the relationship between blood pressure and BPA and phthalate metabolites. Demographic characteristics (age, gender, ethnicity, etc.), prenatal and environmental exposure, study design, measurement methods of urinary metabolites and blood pressure, parameters included in the multivariate analysis may all have an impact on the findings and interpretation.

## Conclusion

Blood pressure profiles were related with BMI-SDS, WBC, platelets, some serum electrolytes (chloride, calcium, ACCa-phosphate product), carotid IMT-SDS, and one of the studied phthalate metabolites (MBzP). MBzP was associated with parental HT, serum albumin and phosphate level, ACCa-phosphate product, and some blood pressure profiles. Interaction between MBzP and blood pressure profiles remained significant after adjusting confounding factors. Further studies in a large sample size with serial measurement are necessary to validate our results.

## Supplementary Information

**Additional file 1**

## Data Availability

Data can be requested from authors via e-mail (S. Songül YALÇIN, siyalcin@hacettepe.edu.tr).

## Abbreviations

ABPM: Ambulatory blood pressure monitoring
ACCa: Albumin-corrected calcium
BMI: Body mass index
BPA: Bisphenol A
fBPA: Free BPA
tBPA: Total BPA
cIMT: Carotid intima media thickness
CKD: Chronic kidney disease
Cre: Creatinine
DBP: Dibutyl phthalate
DEHP: Di (2-ethylhexyl) phthalate
eNOS: Endothelial nitric oxide synthase
eGFR: Estimated glomerular filtration rate
IDH: Isolated daytime hypertension
INH: Isolated nocturnal hypertension
LOD: Limit of detection
MAP: Mean arterial pressure
MnBP: Mono-n-butyl phthalate
MBzP: Mono-benzyl phthalate
MCiNP: Monocarboxy isononyl phthalate
MCiOP: Mono carboxy isooctyl phthalate
MCMHP: Mono 2-carboxymethylhexyl phthalate
MCPP: Mono 3-carboxypropyl phthalate
MECPP: Mono 2-ethyl-5-carboxypentyl phthalate
MEHHP: Mono 2-ethyl 5-hydroxyhexyl phthalate
MEHP: Mono 2-ethylhexyl phthalic acid
MEOHP: Mono 2-ethyl-5-oxy-hexyl phthalate
MEP: Monoethyl phthalate
MiBP: Monoisobutyl phthalate
MiNP: Monoisononyl phthalate
MMP: Monomethyl phthalate
NO: Nitric oxide
OR: Odds ratio
QCL: Quality control low
QCH: Quality control high
SDS: Standard deviation score
ΣDBP: Sum of dibutyl phthalate metabolites
ΣDEHP: Sum of di (2-ethylhexyl) phthalate metabolites
UACR: Urinary albumin to creatinine ratio
UBCR: Urinary beta-2 microglobulin to creatinine ratio
UPCR: Urinary protein to creatinine ratio
WBC: White blood cell
WCHT: White-coat hypertension
