# Supplementary material for: Association of urine phthalate metabolites, bisphenol A levels and serum electrolytes with 24-h blood pressure profile in adolescents
Source: BMC Nephrol. 2022 Apr 12;23:141. doi: 10.1186/s12882-022-02774-y (PMC9004182; doi:10.1186/s12882-022-02774-y)
Supplement: Supplementary file 1 — Additional file 1 [file 12882_2022_2774_MOESM1_ESM.docx]

**Association of urine phthalate metabolites, bisphenol A levels and serum electrolytes with 24-hour blood pressure profile in adolescents**

**Supplementary Tables**

Supplementary Table 1. LOD, LOQ, recovery and quality control analysis of urinary phthalate metabolites

Supplementary Table 2. Distribution of urinary BPA and phthalate metabolites

Supplementary Table 3. Odds ratios for day-time and/or night-time HT diagnosed by ABPM compared to “normal day-time and night-time BP”. comparing detectable or median levels of urinary phthalate metabolites

Supplementary Table 4. The association of the rates of the high ΣDEHP metabolites with z-scores cIMT higher than 95%

**Supplementary Table 1**. LOD, LOQ, recovery and quality control analysis of urinary phthalate metabolites

|  | Molecular weight  (g/mol) | LOD µg/L | LOQ. µg/L | Recovery.  mean | Recovery  SD | QCL  10  mean | QCL  SD | QCL  RSD | QCH  100  mean | QCH  SD | QCH. RSD |
| --- | --- | --- | --- | --- | --- | --- | --- | --- | --- | --- | --- |
| **Mono-benzyl phthalate (MBzP)** | 256.25 | 0.03 | 0.10 | 95.5 | 11.5 | 9.8 | 1.8 | 18.5 | 113.8 | 3.0 | 2.7 |
| **Mono-ethyl phthalate (MEP)** | 194.18 | 0.09 | 0.28 | 98.2 | 3.9 | 9.9 | 0.5 | 4.6 | 107.2 | 7.8 | 7.2 |
| **Mono-(2-ethyl-5-hydroxyhexyl) phthalate (MEHHP)** | 294.34 | 0.06 | 0.17 | 98.1 | 9.9 | 9.4 | 1.2 | 12.8 | 109.5 | 4.8 | 4.4 |
| **Mono-2-ethyl-hexyl phthalate (MEHP)** | 278.34 | 0.30 | 0.89 | 107.5 | 5.6 | 10.5 | 0.2 | 1.8 | 109.8 | 4.9 | 4.5 |
| **Mono-carboxyisononyl phthalate (MCiNP)** | 336.38 | 0.09 | 0.28 | 99.6 | 12.7 | 10.9 | 0.8 | 6.9 | 108.5 | 2.5 | 2.3 |
| **Mono-carboxyisooctyl phthalate (MCiOP)** | 322.35 | 0.06 | 0.18 | 87.3 | 4.4 | 11.4 | 0.4 | 3.8 | 111.1 | 3.0 | 2.7 |
| **Mono-(2-ethyl-5-carboxypentyl) phthalate (MECPP)** | 308.33 | 0.27 | 0.80 | 103.2 | 11.8 | 9.6 | 0.9 | 9.6 | 107.8 | 5.9 | 5.4 |
| **Mono-n-butyl phthalate (MnBP)** | 222.24 | 0.09 | 0.26 | 84.7 | 11.1 | 10.5 | 0.8 | 7.6 | 87.1 | 2.6 | 3.0 |
| **Mono-(3-carboxypropyl) phthalate (MCPP)** | 252.22 | 0.09 | 0.28 | 98.0 | 9.4 | 9.1 | 0.5 | 5.5 | 107.0 | 6.9 | 6.5 |
| **Mono-n-methyl phthalate (MMP)** | 180.16 | 0.07 | 0.21 | 93.4 | 9.6 | 10.3 | 2.1 | 20.0 | 102.4 | 3.9 | 3.8 |
| **Mono-isononyl phthalate (MNP)** | 292.37 | 0.16 | 0.49 | 112.8 | 4.4 | 10.6 | 0.9 | 8.2 | 109.4 | 4.3 | 3.9 |
| **Mono-(2-ethyl-5-oxohexyl) phthalate (MEOHP)** | 292.33 | 0.15 | 0.45 | 105.3 | 9.8 | 10.7 | 0.6 | 5.4 | 101.7 | 3.0 | 3.0 |
| **Mono-isobutyl phthalate (MiBP)** | 222.24 | 0.07 | 0.22 | 90.0 | 11.0 | 10.3 | 0.7 | 6.4 | 94.6 | 3.3 | 3.4 |

**Supplementary Table 2.** Distribution of urinary bisphenol A (BPA) and phthalate metabolites

|  |  |  | Percentile | | | | |  |
| --- | --- | --- | --- | --- | --- | --- | --- | --- |
|  |  | Mean | 10th | 25th | 50th | 75th | 90th | <DL.% |
| Total BPA | µg/L | 141.4 | 63.1 | 91.8 | 142.0 | 181.9 | 216.0 | 0.0 |
|  | µg/g-cr | 113.0 | 33.2 | 52.6 | 96.7 | 152.7 | 223.4 |  |
| Free BPA | pg/mL | 5.5 | <DL | <DL | <DL | <DL | 34.3 | 87.2 |
| MBzP | µg/L | 1.00 | <DL | <DL | <DL | 0.18 | 4.18 | 75.6 |
|  | µg/g-cr | 0.81 | <DL | <DL | <DL | 0.05 | 3.18 |  |
| MEP | µg/L | 51.7 | 3.1 | 8.5 | 20.6 | 49.7 | 131.3 | 4.7 |
|  | µg/g-cr | 42.3 | 2.6 | 5.3 | 12.7 | 45.7 | 102.7 |  |
| MEHHP | µg/L | 59.4 | 19.8 | 30.3 | 52.9 | 80.5 | 104.0 | 0.0 |
|  | µg/g-cr | 44.4 | 12.9 | 22.2 | 38.3 | 54.4 | 78.2 |  |
| MEHP | µg/L | 7.8 | 1.6 | 3.7 | 5.8 | 9.2 | 15.6 | 2.3 |
|  | µg/g-cr | 6.1 | 1.2 | 2.5 | 4.2 | 7.0 | 14.2 |  |
| MCiNP | µg/L | 0.97 | <DL | 0.35 | 0.87 | 1.45 | 1.92 | 22.1 |
|  | µg/g-cr | 0.69 | <DL | 0.19 | 0.59 | 0.96 | 1.57 |  |
| MCiOP | µg/L | 6.0 | 0.9 | 1.5 | 2.4 | 4.7 | 7.8 | 0.0 |
|  | µg/g-cr | 3.9 | 0.7 | 1.1 | 1.8 | 3.2 | 6.5 |  |
| MECPP | µg/L | 17.6 | 6.2 | 8.3 | 15.1 | 24.9 | 33.2 | 0.0 |
|  | µg/g-cr | 13.5 | 3.9 | 6.4 | 10.4 | 17.6 | 23.4 |  |
| MnBP | µg/L | 25.1 | 10.1 | 14.6 | 20.0 | 32.3 | 47.2 | 0.0 |
|  | µg/g-cr | 20.2 | 6.5 | 9.6 | 13.6 | 22.9 | 33.8 |  |
| MCPP | µg/L | 0.65 | <DL | 0.26 | 0.50 | 1.11 | 1.52 | 19.8 |
|  | µg/g-cr | 0.50 | <DL | 0.13 | 0.34 | 0.67 | 1.36 |  |
| MMP | µg/L | 5.6 | <DL | 1.0 | 2.6 | 6.3 | 15.1 | 10.5 |
|  | µg/g-cr | 4.8 | <DL | 0.6 | 1.8 | 4.8 | 13.7 |  |
| MiNP | µg/L | 0.50 | <DL | <DL | <DL | <DL | 1.50 | 88.4 |
|  | µg/g-cr | 0.41 | <DL | <DL | <DL | <DL | 1.20 |  |
| MEOHP | µg/L | 13.1 | 3.9 | 6.7 | 10.6 | 16.5 | 24.9 | 0.0 |
|  | µg/g-cr | 10.0 | 1.9 | 5.2 | 8.1 | 11.0 | 17.7 |  |
| MiBP | µg/L | 26.8 | 13.0 | 15.7 | 20.8 | 33.0 | 50.6 | 0.0 |
|  | µg/g-cr | 21.8 | 6.3 | 10.4 | 14.7 | 24.3 | 35.9 |  |
| MCMHP | µg/L | 8.4 | 2.2 | 3.8 | 6.0 | 8.6 | 14.4 | 0.0 |
|  | µg/g-cr | 5.9 | 1.6 | 2.8 | 4.3 | 6.6 | 9.7 |  |
| DEHP; primary/secondary | ratio | 8.5 | 3.0 | 4.6 | 6.9 | 10.2 | 16.6 |  |
| ΣDEHP metabolites | µg/L | 108.1 | 40.6 | 59.5 | 94.5 | 143.8 | 186.1 |  |
|  | µg/g-cr | 82.5 | 25.7 | 44.9 | 69.1 | 102.6 | 136.6 |  |
| ΣDBP metabolites | µg/L | 51.9 | 23.3 | 30.7 | 40.7 | 62.7 | 96.9 |  |
|  | µg/g-cr | 41.9 | 14.2 | 20.6 | 28.7 | 46.8 | 69.3 |  |

MnBP: mono-n-butyl phthalate, MBzP: mono-benzyl phthalate, MCiNP: monocarboxy isononyl phthalate, MCiOP: mono carboxy isooctyl phthalate, MCMHP: mono 2-carboxymethylhexyl phthalate, MCPP: mono 3-carboxypropyl phthalate, MECPP: mono 2-ethyl-5-carboxypentyl phthalate, MEHHP: mono 2-ethyl 5-hydroxyhexyl phthalate, MEHP: mono 2-ethylhexyl phthalic acid, MEOHP: mono 2-ethyl-5-oxy-hexyl phthalate, MEP: monoethyl phthalate, MiBP: monoisobutyl phthalate, MiNP: monoisononyl phthalate, MMP: monomethyl phthalate, ΣDBP metabolites: sum of dibutyl phthalate metabolites, ΣDEHP metabolites: sum of di (2-ethylhexyl) phthalate metabolites.

**Supplementary Table 3**. Odds ratios for day-time and/or night-time HT diagnosed by ABPM compared to “normal day-time and night-time blood pressure” (comparing detectable or median levels of urinary phthalate metabolites)

|  |  | OR (95% CI) | AOR (95% CI)* | AOR (95% CI)** | AOR (95% CI)*** |
| --- | --- | --- | --- | --- | --- |
| MCiNP | ≥0.9 vs. <0.9 µg/L | 0.81 (0.32-2.07) | 0.67 (0.23-1.91) | 0.56 (0.18-1.70) |  |
| MEHHP | ≥50 vs. <50 µg/L | 2.50 (0.94-6.67) | 2.66 (0.86-8.21) | 2.63 (0.84-8.22) | 2.39 (0.66-8.67) |
| DEHP primary/secondary ratio | ≥6.9 vs. <6.9 | 0.45 (0.17-1.17) | 0.65 (0.23-1.87) | 0.73 (0.25-2.11) |  |
| MBzP | ≥DL vs. <DL | **5.33 (1.85-15.34)** | **6.15 (1.68-22.59)** | **6.39 (1.70-23.99)** | **6.38 (1.54-26.44)** |
| MiBP | >20 vs. ≤20 µg/L | 1.55 (0.60-3.99) | 2.15 (0.73-6.31) | 2.02 (0.68-6.03) |  |
| MEP | ≥20 vs. <20 µg/L | 2.10 (0.80-5.50) | 2.71 (0.90-8.18) | 2.69 (0.88-8.19) | 3.37 (0.98-11.64) |
|  |  |  |  |  |  |
| MEP | MBzP |  |  |  |  |
| ≥20 µg/L | ≥DL | 12.25 (2.43-61.56) | 20.10 (2.59-156.27) | 19.14 (2.47-148.08) |  |
| <20 µg/L | ≥DL | 7.00 (1.38-35.48) | 10.92 (1.57-75.75) | 10.84 (1.50-78.30) |  |
| ≥20 µg/L | <DL | 2.63 (0.72-9.61) | 4.19 (0.95-18.54) | 3.93 (0.88-17.48) |  |
| <20 µg/L | <DL | 1.00 | 1.00 | 1.00 |  |

* adjusted for age, sex, BMI-SDS, parental hypertension

** adjusted for age, sex, BMI-SDS, parental hypertension, platelet, eGFR, urinary creatinine

*** adjusted for age, sex, BMI-SDS, parental hypertension, platelet. eGFR, urinary creatinine, selected three urinary phthalate metabolites

BMI-SDS: body mass index standard deviation score, eGFR: estimated glomerular filtration rate, DEHP: di (2-ethylhexyl) phthalate, MBzP: mono-benzyl phthalate, MCiNP: monocarboxy isononyl phthalate, MEHHP: mono 2-ethyl 5-hydroxyhexyl phthalate, MEP: monoethyl phthalate, MiBP: monoisobutyl phthalate.

**Supplementary Table 4.** The association of the rates of the high ΣDEHP metabolites with cIMT-SDS higher than 95th percentile

|  |  | OR (95% CI) | AOR (95% CI)* | AOR (95% CI)** |
| --- | --- | --- | --- | --- |
| ΣDEHP metabolites | ≥100 vs. <100 µg/L | 2.68 (1.08-6.66) | 2.90 (1.12-7.49) | 3.05 (1.16-8.02) |

*adjusted for age, sex, BMI-SDS

**adjusted for age, sex, BMI-SDS, eGFR. urinary creatinine

BMI-SDS: body mass index standard deviation score, cIMT-SDS: carotid intima media thickness, eGFR: estimated glomerular filtration rate

ΣDEHP metabolites: sum of di (2-ethylhexyl) phthalate metabolites.
